# Tuberculous Tenosynovitis Presenting as Ganglion of Wrist

**DOI:** 10.1155/2012/143921

**Published:** 2012-12-24

**Authors:** Shahaji Chavan, Shyamsunder Shambhu Sable, Sachin Tekade, Prashant Punia

**Affiliations:** Department of General Surgery, Padmashree Dr. D. Y. Patil Medical College, Pimpri, Pune 411018, India

## Abstract

Tuberculosis (TB) is still endemic in many developed countries. Involvement of the hand and wrist at presentation is extremely rare, and the diagnosis is often missed. A 57 years old male presented with swelling over the left wrist since 3 years Three swellings over dorsal aspect of the left wrist Soft in consistency Non tender Non compressible Mobile at right angles to the plane of the wrist joint. ESR: 45 mm in 1 hr and rest blood investigations were normal. Ultrsonography showed giant cell tumor of Extensor Digitorum sheath. X-ray: soft tissue swelling and MRI was suggestive of extensor tendon sheath extraskeletal synovial Koch's, or giant cell tumor of tendon sheath. Excision of swelling was planned and intraoperatively, rice bodies were seen inside it. Histopathological examination showed caseous necrosis with granuloma formation. Patient was put on DOT1 therapy. Tuberculous tenosynovitis was first described by Acrel in 1777. Rice bodies occurring in joints affected by tuberculosis were first described in 1895 by Reise. Rice bodies will be diagnosed on plain radiographs when mineralization occurs. More than 50% of cases recur within 1 year of treatment. The currently recommended 6-month course is often adequate with extensive curettage lavage and synovectomy should be performed. Surgery is essential, but the extent of surgical debridement is still debatable. The surgeon has to be aware of the significance of loose bodies when performing routine excision of innocuous looking wrist ganglia.

## 1. Introduction

Tuberculosis (TB) is still endemic in many developed countries. Involvement of the hand and wrist at presentation is extremely rare, and the diagnosis is often missed. Extrapulmonary tuberculosis involvement of the musculoskeletal system is uncommon, accounting for only 10% of tuberculosis (TB) cases.

## 2. Case Report 

57-years-old male presented with swelling over the left wrist since 3 years. No history of trauma, fever. Past history: no history of tuberculosis, diabetes, and hypertension. 

### 2.1. General Examination

Pulse-78 beat/min BP-128/70 mmhg.

No pallor, cyanosis, clubbing, edema, and lymphadenopathy

### 2.2. Local Examination

Three swellings over dorsal aspect of the left wrist: soft in consistency, nontender, noncompressible, and mobile at right angles to the plane of the wrist joint.

### 2.3. Systemic Examination

 Respiratory system: AEBE.
 Cardiovascular system: S1 S2 present.
 Abdominal examination: NAD.
 Central nervous system: NAD.

## 3. Investigations

 Hb: 12 gm%, TLC: 8600/cmm, P: 65%, L: 20%, M: 03%, and E: 01%
 ESR: 45 mm in 1 hr, Bsl (R): 95 mg%, BT: 01 min 5 sec, and CT: 04 min 10 sec,
 BUN: 25 mg%, S.creat: 0.8 mg%, and USG: Giant-cell tumor of Extensor Digitorum sheath.
 X-ray: soft tissue swelling, MRI: extensor tendon sheath, extraskeletal synovial Koch's, or giant cell tumor of tendon sheath.

### 3.1. Intraoperative Finding of Rice Bodies

Histopathological examination showed caseous necrosis with granuloma formation. Patient was started on DOTS Category I and stitches were removed on postoperative day 8.

## 4. Discussion

**Figure 1:**
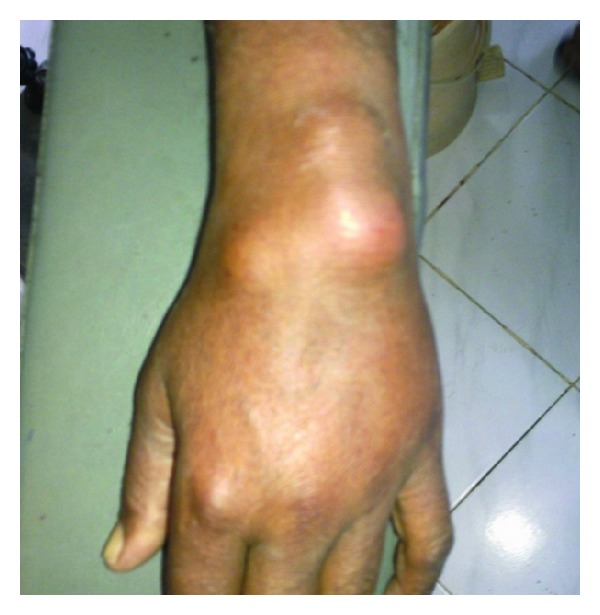


**Figure 2:**
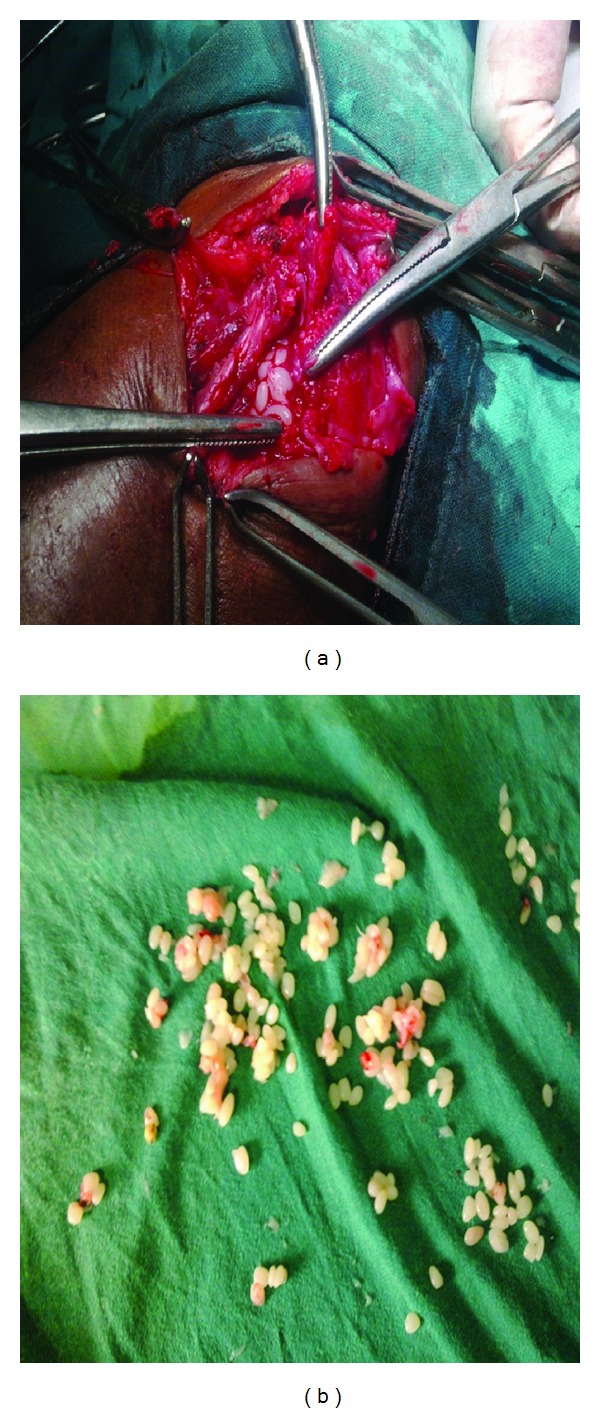


Tuberculous tenosynovitis was first described by Acrel in 1777 [1]. Rice bodies occurring in joints affected by tuberculosis were first described in 1895 by Reise [2]. Rice bodies are a common finding in many rheumatic diseases such as rheumatoid arthritis, systemic lupus erythematosus, seronegative arthritis, nonspecific arthritis, tuberculosis, atypical mycobacterial infections, and osteoarthritic joints (Figure 2) [3]. The sheath of the tendons of the wrist and hand has been reported as a site for rice body formation (Figure 1). Rice bodies will be diagnosed on plain radiographs when mineralization occurs [4]. MRI showed thickening of the synovial membrane with increased vascularization, fluid within the tendon sheath, reactive inflammation around the tendon, or swelling of the tendon [5]. Tendon is replaced by vascular granulation tissue. Sheath is obliterated by fibrous tissue, fluid is confined within the sheath, and rice bodies form due to caseation, and Tendon may consist of only a few strands of tissue and may rupture spontaneously [1]. More than 50% of cases recur within 1 year of treatment [6]. The currently recommended 6-month course is often adequate with Extensive curettage, lavage and synovectomy should be performed. Surgery is essential, but the extent of surgical debridement is still debatable [7].

## 5. Conclusion

Tuberculous tenosynovitis of wrist is rare and treatment comprises of excision of lesion and antituberculous chemotherapy will minimize recurrence of disease. 

The surgeon has to be aware of the significance of loose bodies when performing routine excision of innocuous looking wrist ganglia.

## References

[1] Aboudola S, Sienko A, Carey R, Johnson S (2004). Tuberculous tenosynovitis. *Human Pathology*.

[2] Reise H (1895). Die Reiskorpschen in tuberculserkrankensynovalsacken. *Deutsche Zeitschrift für Chirurgie*.

[3] Chau CLF, Griffith JF, Chan PT, Lui TH, Yu KS, Ngai WK (2003). Rice-body formation in atypical mycobacterial tenosynovitis and bursitis: findings on sonography and MR imaging. *American Journal of Roentgenology*.

[4] Griffith JF, Peh WCG, Evans NS, Smallman LA, Wong RWS, Thomas AMC (1996). Multiple rice body formation in chronic subacromial/subdeltoid bursitis: MR appearances. *Brain and Language*.

[5] Hoffman KL, Bergman AG, Hoffman DK, Harris DP (1996). Tuberculous tenosynovitis of the flexor tendons of the wrist: MR imaging with pathologic correlation. *Skeletal Radiology*.

[6] Garrido G, Gomez-Reino JJ, Fernández-Dapica P, Palenque E, Prieto S (1988). A review of peripheral tuberculous arthritis. *Seminars in Arthritis and Rheumatism*.

[7] Pimm LH, Waugh W (1957). Tuberculous tenosynovitis. *The Journal of Bone and Joint Surgery*.

